# Influencing factors and mechanism of high-speed railway passenger overall comfort: Insights from source functional brain network and subjective report

**DOI:** 10.3389/fpubh.2022.993172

**Published:** 2022-09-23

**Authors:** Chaojie Fan, Yating Lin, Shuxiang Lin, Yingli Li, Fan Wu, Xiaohui Xiong, Wei Zhou, Dan Zhou, Yong Peng

**Affiliations:** ^1^Key Laboratory of Traffic Safety on Track of Ministry of Education, School of Traffic and Transportation Engineering, Central South University, Changsha, China; ^2^Joint International Research Laboratory of Key Technology for Rail Traffic Safety, School of Traffic and Transportation Engineering, Central South University, Changsha, China

**Keywords:** high-speed railway, passenger overall comfort, railway worker health, subjective report, source functional brain network

## Abstract

Overall comfort is the priority for the high-speed railway (HSR) passengers, while its influencing factors and mechanism are not yet apparent. According to the source functional brain network and subjective report, this study revealed the potential influencing factors and mechanisms of passengers overall comfort in high-speed railway environments. Here, an ergonomics field test with 20 subjects was conducted where subjective reports and electroencephalography (EEG) were collected. The electric-source imaging and functional connectivity were used to build the source functional brain network from EEG and network indices were extracted. Statistics analysis results showed that static comfort played the most critical role in the overall comfort, followed by emotional valence, emotional arousal, aural pressure comfort, vibration comfort, and noise comfort. Thermal and visual comfort were insignificant due to the well-designed heating, ventilation, and air conditioning (HVAC) and lighting system of HSR. In addition, the source functional brain network of passengers who felt uncomfortable had the higher clustering coefficient, assortativity coefficient and global efficiency, which meant greater activation of brain compared with passengers who were in a state of comfort. According to the local attributes indices analysis, most key brain regions were located in the frontal and hippocampus, which revealed emotion and spatial perception contribute to the whole comfort degradation process. This work proposed novel insights into HSR passengers overall comfort according to subjective and objective methods. Our findings demonstrate emotional regulation and seat improvements are key factors for future improvement of HSR passengers overall comfort.

## 1. Introduction

As one of the core services provided by high-speed railways (HSR), passenger comfort is a concrete manifestation of advanced technologies and a critical goal of the design process for HSR (1–3). Improving the comfort of passengers is the goal of HSR development worldwide. What is even more noteworthy is that the overall comfort of HSR has a significant impact on the railway employees such as the drivers and the conductors (4). Long-term uncomfortable conditions can pose challenges to the occupational health of railway employees, particularly their mental health. However, the complex environment of HSR and the narrow and closed compartments pose many challenges to the overall comfort of passengers.

Peng et al. summarized the overall comfort of high-speed trains into six aspects based on the sources of discomfort: static comfort, vibration comfort, noise comfort, thermal comfort, aural pressure comfort, and visual comfort (5). Specifically, static comfort is mainly due to inappropriate seat design and passenger discomfort caused by the cabin environment. Vibration comfort is reflected in the discomfort induced by vibrations transmitted to passengers at the seat back and cushion in complex operating environments (6). The aerodynamic, wheel-track, and traction noise generated during train operation gradually becomes more pronounced and leads to serious noise comfort problems (7). When high-speed trains pass through tunnels at high speed, changes in pressure inside the car can cause different degrees of ear pain or tinnitus and other ear pressure discomfort (8, 9). The thermal environment within the relatively confined passenger compartments of high-speed trains relies heavily on air conditioning and ventilation systems to vary and maintain, and an unsuitable thermal environment can lead to thermal comfort problems (10). Changes in light and darkness in the cabins can affect the visual comfort of passengers, especially when HSR through tunnels at high speed (11). In addition, the impact of passengers' own emotions on comfort has received increasing scholarly attention in recent years (12, 13).

To sum up, there are many factors that affect the overall comfort of high-speed train passengers, and the mechanism of comfort deterioration is not yet clear. It is currently a major issue in the field of high-speed train research. An accurate analysis of influencing factors and mechanism is the key to improve the overall comfort of HSR passengers. Therefore, there is an urgent need to conduct research on the overall comfort of HSR passengers in real-life environments, and to use subjective report and golden objective evaluation indicators such as EEG signals to reveal such factors and mechanism.

### 1.1. Related works

#### 1.1.1. Subjective indicators

Generally speaking, comfort is a subjective feeling in the human body. When passengers are comfortable, they should be content with the conditions of their surroundings, which means they should feel satisfied with their surroundings. It also means they are relaxed, stretched, happy, and in harmony with the outside world, and with little discomfort (5, 14). Therefore, subjective assessment is the most common method of comfort assessment and includes most of the internationally accepted comfort standards, such as ISO 2631-1: 1997 (15), UIC 553:2004 (16), UIC 660:2002 (17). They were all developed using subjective assessments as criteria for setting environmental parameter thresholds. However, most of the related research has been carried out in the laboratory or driving simulators, which could only simulate one or two environmental parameters (18–22). The complex environment in HSR and the cross-fertilization of all factors makes it challenging to analyse influencing factors of passengers overall comfort according to subjective indicators in the laboratory. The influencing factors of HSR passenger overall comfort have not been fully characterized.

#### 1.1.2. Objective indicators

According to the type of indicators, we separated the objective indicator into human parameters and environmental parameters. Environmental indicators are mostly standards pointing out, for example, vibration amplitude and frequency related to vibration comfort, sound pressure level related to noise comfort, temperature and humidity related to thermal comfort, luminous flux related to visual comfort, etc. (5). These standards were developed by considering only the role of individual factors and did not consider the coupling effect between environmental factors. New ways of evaluating human factors that offer insight into human physiology and biomechanics have been made possible by developments in sensors and computer numerical simulation technologies. Interface pressure (23, 24) skin parameter (25), muscle, and skeleton force (26), peripheral nervous signal like electromyogram (EMG) (27), electrocardiograph (ECG) (28), galvanic skin response (GSR) (29) etc., and central nervous signal like electrophotography(EEG) (30–32) have been widely used in the present review. Among them, EEG was rewarded as “golden standard” because comfort is by nature a sensation derived from the brain (13).

#### 1.1.3. EEG-based methods

As EEG is one of the best objective criteria for evaluating comfort, many scholars have used EEG in recent years to study the influencing factors and mechanisms of comfort (13, 32, 33). For example, Fukai et al. proposed a method to assess the ride comfort of cars with different tires using EEG (34). Shan et al. used different classifiers to classify the real-time thermal comfort state of passengers and achieved good classification results (35). EEG is regarded as the gold standard for evaluating human comfort recently.

Despite its long success, current EEG-based methods in the field of comfort analysis have two main problems. The first one is the spatial resolution of traditional EEG is limited, which is mainly caused by the volume conduction effect. EEG can not reflect the real activity of cerebral cortex. When passing through nerve tissue, cerebrospinal fluid, meninges, low conduction skull and scalp, the potential generated by nerve source is weakened and blurred. Advanced EEG imaging technology is needed to compensate the brain volume conduction effect and improve the spatial resolution of EEG (36, 37). The second one is ignoring the brain connectivity during the whole analyzing process. Modern neuroscience research proposed that comparing the specific brain region brain signal, the connectivity between different brain regions are more important. Abnormal feelings such as anxiety and discomfort are, to some extent, due to strange connections between the brain areas involved (38).

### 1.2. Contributions

To fill the research gap mentioned above, in this study, a field ergonomics test in the HSR of China was carried out. The subjective reports of 20 passengers were obtained and the source functional brain network were calculated according to their EEG, which are used to reveals the influencing factors and mechanisms of HSR passenger overall comfort. The main contributions of this study can be summarized as follow:

First, a filed ergonomics test was carried out, which can help us obtain the real feedback of HSR passengers. Second, the statistically significant influencing factors of HSR passengers overall comfort were confirmed by using the subjective reports.

Third, influencing mechanism of HSR passengers overall comfort were likely revealed according to attribute indices calculated source functional brain network.

## 2. Experiment procedures

### 2.1. Subjects

Twenty healthy right-handed adults were recruited for our tests, including eleven males and nine females (23.2 ± 1.8 year old). To make the experiment more accurate and reduce the influence of extraneous variables on the experiment, each subject participated on the same frequency of high-speed trains at the same time of the day. They did not receive medical treatment for the disease before participating in the experiment. Drugs and alcohol were prohibited 24 h before the experiment. Before every participant took part in the test, they were informed of the purpose, the procedure of the test, and the harmlessness of the EEG signal acquisition equipment. This project was approved by Xiangya No.2 Hospital of Central South University Institutional Review Board.

#### 2.1.1. Apparatus

**Figure 1:**
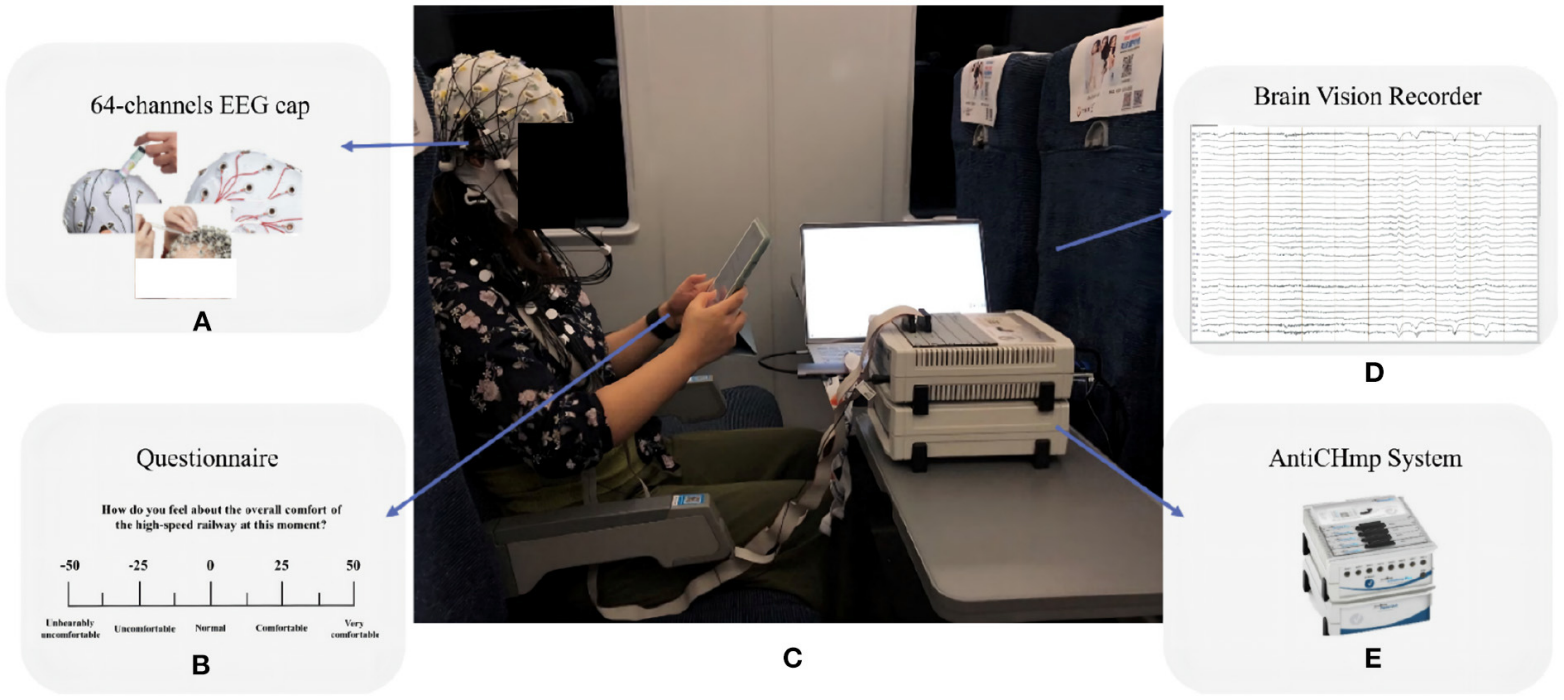
On-vehicle experiment scene. **(A)** 64-channels EEG cap. **(B)** Questionnaire. **(C)** Experiment scene. **(D)** Brain Vision recorder. **(E)** AntiCHmp system.

The test equipment used in this trial is the BP actiCHamp EEG acquisition system (Figure 1E), which is equipped with a 64-channel EEG amplifier (Figure 1A), an EEG signal recording and analysis software. The system has a maximum sampling rate of 50 kHz and the EEG electrodes were placed according to the international 10-20 system.

### 2.2. Procedure

This experiment was carried out with the support of the China Railway Guangzhou Group Co., Ltd. The route of the experiment was the Changsha-Guiyang section of the Shanghai-Kunming line to stimulate discomfort among subjects (Figure 1C). To minimize the interference of extraneous factors, every subject took a second-class B seat (i.e., the middle of the triple seat), as shown in Figure 1. The test hours, train number, and environmental conditions around the test passenger remained consistent throughout the test. The speed of the HSR was maintained at around 300 km/h and each trail took approximately 160 min.

In the experiment, EEG data were recorded from the high-speed train starting running to the end at a sampling rate of 500 Hz, and the subjects were asked to complete a subjective questionnaire on an iPad every 10 min until arriving at the terminus of the experiment. During EEG recording, data were band-pass filtered between 0.01 and 40 Hz and processed through a 50 Hz notch filter with impedance kept below 5 k.

### 2.3. Subjective questionnaire design

In general, the comfort of high-speed train passengers is not only related to objective factors such as environmental parameters but also affected by subjective factors such as physiological changes and psychological changes. In this paper, the subjective questionnaire method is used to evaluate the indoor environmental parameters such as vibration, noise, air pressure, light, temperature and the emotional state of the HSR passengers through real-world tests. The subjective questionnaire items are listed in Table 1.

**Table 1:** Subjective questionnaire.

**Question no**.	**Questions**
1	Overall comfort
2	Emotion value
3	Emotion arousal
4	Static comfort
5	Noise comfort
6	Vibration comfort
7	Aural pressure comfort
8	Visual comfort
9	Thermal comfort

The questionnaire was designed using a visual analog scale approach. It contains questions related to the comfort of the high-speed train riding environment, including the subjective perception of the comfort-related question. For example, “How do you feel about the overall comfort of the high-speed train at this time?”. As shown in Figure 1B, the questionnaire is rated on a scale from −50 to 50, where −50 is “very uncomfortable”, −25 is “uncomfortable”, 0 is “neutral”, 25 being “comfortable”, and 50 being “very comfortable”.

## 3. Functional brain network analysis

**Figure 2:**
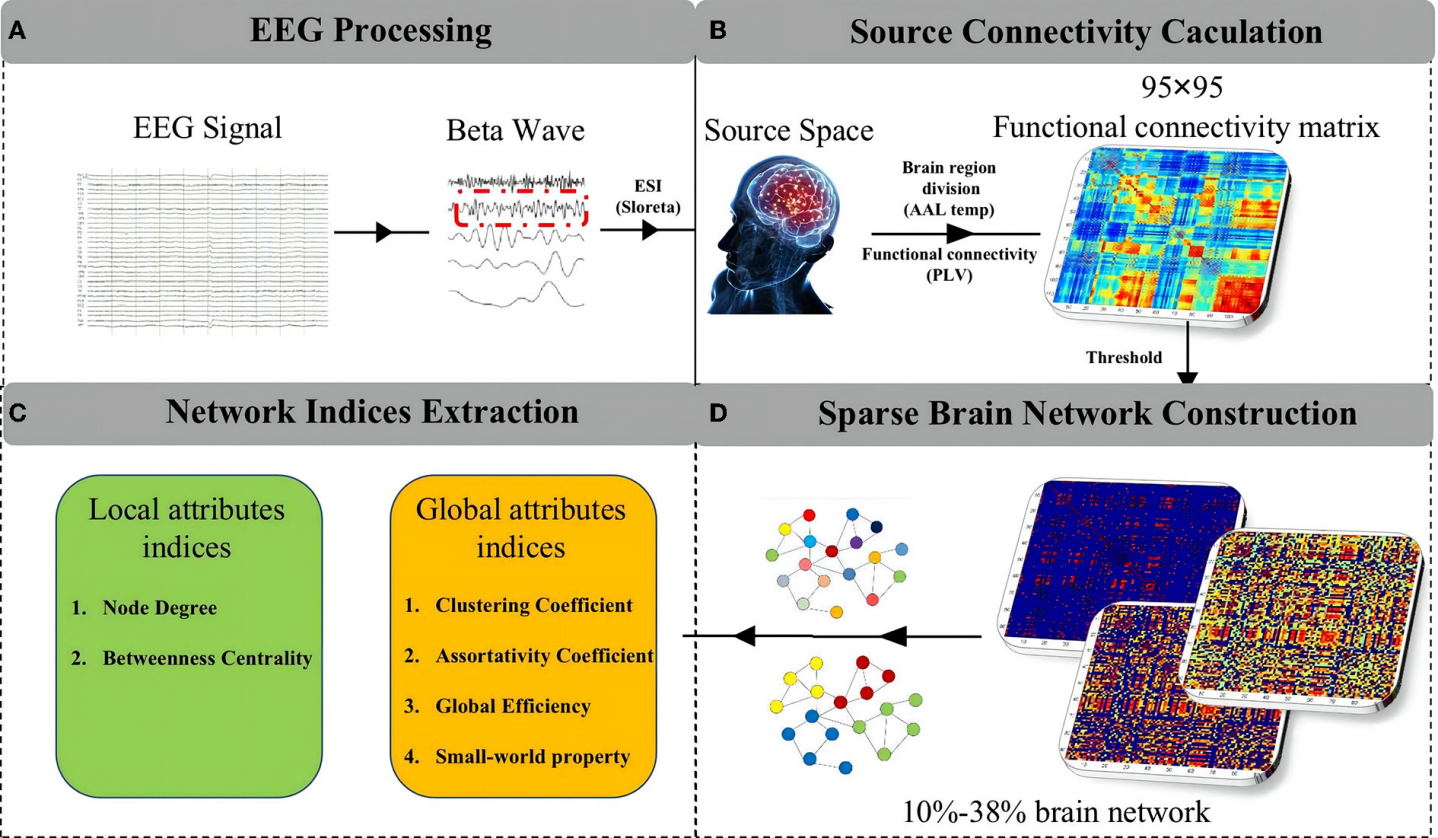
The detailed procedure of functional brain network analysis. **(A)** EEG processing. **(B)** Source connectivity calculation. **(C)** Network indices extraction. **(D)** Sparse brain network construction.

The detailed procedure of functional brain network analysis can be summarized in four steps, which are described by the detailed flowchart shown in Figure 2. The framework consists of four steps: EEG processing, source connectivity calculation, sparse brain network construction, and network indices extraction.

### 3.1. EEG processing

#### 3.1.1. EEG preprocessing

The whole EEG preprocessing steps were similar to our previous research (13), which included EEG re-referencing and re-sampling, artifacts removing and data dividing. The wavelet packet transform was used to extract the five frequency bands (delta: 1–3 Hz, theta: 4–7 Hz, alpha: 8–13 Hz, beta: 14–30 Hz, gamma: 31–64 Hz) from the EEG data (32). In our previous work, we have identified the primary comfort-related rhythmic wave as the beta band. Therefore, our research in this paper focuses on the EEG signal of the beta wave. In addition, based on the results of the subjective overall comfort evaluation, we extracted two groups from the total EEG data, the comfortable and uncomfortable groups.

#### 3.1.2. Electric-source imaging

To localize the source activity of the brain, the inverse problem is usually computed for EEG signals, i.e., the equivalent dipole or current density on a predefined surface or 3D volume is estimated under a priori assumptions (37). Due to the non-uniqueness of the inverse problem, most researchers have proposed some common constraint-based source reconstruction algorithms, including Partial Cannonical Correlation (PCC) (39), Minimum Norm Estimation (MNE) (40), and Standardized Low-resolution Electromagnetic Tomography (sLORETA) (41). Among them, sLORETA works well under noise conditions, and thus is adopted as the ESI method in this paper. This method is based on the assumption that the current density at any point of the cerebral cortex is very close to the average current density in its vicinity, with Laplace constraints attached to Weighted Minimum Norm Estimation (WMNE).

### 3.2. Source connectivity calculation

The core of network are nodes and edges and the nodes of our brain network are brain regions. Nowadays, the anatomical automatic labeling (AAL) template provided by the MNI agency with 95 regions leads to the majority of existing functional brain network studies using them (42). Therefore, in this paper, AAl template was used as the standard for brain region delineation so that the number of nodes is 95.

The edges of brain source connectivity networks are connectivity relationships between brain nodes, including structural connectivity and functional connectivity. The former is based on spatial location and the latter on the correlation of information in brain regions. In this paper, Phase Locking Value (PLV) was selected as the functional connectivity indicator to calculate nodal correlation (43). PLV is the absolute value of the average phase difference between two signals and is used by many researchers to measure the degree of phase synchronization of inter-lead EEG signals in a narrow frequency band (44). The calculation formula of PLV was shown in below:


(1) $$PLV\left(t\right)=\frac{1}{N}\left|\sum_{n=1}^{N}\exp\left(j\left(\Delta \varphi_{n}\left(t\right)\right)\right)\right|$$

where Δφ_*n*_(*t*) = (φ_*x*_(*t*)−φ_*y*_(*t*)) means the phase difference between the signal *x* and the signal *y* at time *t*.

### 3.3. Sparse brain network construction

After steps above, A power-free graph was constructed based on fully connected functional connectivity matrix of each subjects. And a sparse operation was applied in all fully connected functional connectivity matrices by setting different sparse density. This paper uses a new sparsity range determination method proposed by (45). The sparsity range was determined to be 10–38% and eight functional brain networks were created at each sparsity level, i.e., 10, 14, 18, 22, 26, 30, 34, and 38%, when a growth step of 4% was chosen. In addition, in order to comprehensively compare the differences between functional brain networks of different sparsity, AUC was used as an overall evaluation index.

### 3.4. Network indices extraction

In order to describe the differences and variations in functional brain networks, this paper extracts some indices of brain network properties and uses them to describe and analyse brain networks of passengers at different comfort states. These indices can be divided into two categories, one for global attributes and the other for local attributes.

#### 3.4.1. Global attributes index

##### 3.4.1.1. Clustering coefficient

The clustering coefficient is often used to measure the degree of grouping and tightness of brain node connections within a functional brain network (46). Larger clustering coefficients indicate stronger association of brain node groups in functional brain networks. The clustering coefficient can be found according to Equation (2).


(2) $$CC=\frac{1}{n}\sum i\in N^{\frac{2t_{i}^{w}}{k_{i}(k_{i})}}$$

##### 3.4.1.2. Assortativity coefficient (AC)

The assortativity coefficient is often used to quantify the adaptability of large functional brain networks (47). Assortativity is the tendency of a node to connect to nodes similar to it, while the opposite tendency is called heterogeneity. The calculation formula is shown in equation below:


(3) $$AC=\frac{M^{-1}\sum_{i}j_{i}k_{i}-[M^{-1}\sum_{i}\frac{1}{2}(j_{i}^{2}+k_{i}^{2}){]}^{2}}{M^{-1}\sum_{i}\frac{1}{2}(j_{i}^{2}+k_{i}^{2})-{\left[M^{-1}\sum_{i}\frac{1}{2}(j_{i}^{2}+k_{i}^{2})\right]}^{2}}$$

##### 3.4.1.3. Global efficiency (GE)

Global efficiency is a measure of the efficiency of information exchange in functional brain networks and is defined as Equation (4) (48). Global efficiency is the average of the reciprocal of the shortest paths in a functional brain network. Generally speaking, the global efficiency is The higher the global efficiency and the shorter the shortest path length, the faster the rate of information transfer between brain nodes in the functional brain network.


(4) $$GE=\frac{1}{n}\sum i\in N^{\frac{\sum j\in N,j\neq i^{{\left(d_{ij}^{w}\right)}^{-1}}}{n-1}}$$

##### 3.4.1.4. Small-world property (σ)

A small-world network is a graph consisting of a large number of vertices, where the average path length between any two points is much smaller than the number of vertices (49). Small world property (σ) are often used to quantitatively represent the small-world network characteristics of functional brain networks.


(5) $$\begin{array}{l} \sigma =\frac{\frac{CC_{real}}{CC_{random}}}{\frac{CPL_{real}}{CPL_{random}}} \end{array}$$

where *CC*_*real*_ and *CC*_*random*_ denote the clustering coefficients of the actual constructed functional brain network and the clustering coefficients of the random network, respectively, and *CPL*_*real*_ and *CPL*_*random*_ denote the feature path length and feature path length of the actual constructed functional brain network, respectively.

#### 3.4.2. Local attributes index

##### 3.4.2.1. Node degree (Deg)

Nodal Degrees is the most important basic metric property in functional brain networks (50). The node degree is the number of edges associated with that node. A larger degree of a brain node means that the brain node is more important.


(6) $$\begin{array}{l} Deg_{i}=k_{i} \end{array}$$

where *k*_*i*_ is the number of the edges which connected to the node *i*.

##### 3.4.2.2. Betweenness centrality (BC)

The number of shortest paths through a brain node in a functional brain network is defined as betweenness centrality (51).


(7) $$\begin{array}{l} BC_{i}=\underset{s\neq i\neq t}{\sum }\frac{n_{st}^{i}}{g_{st}} \end{array}$$

where $n_{st}^{i}$ denotes the number of paths that pass through node *i* and are shortest paths, *g*_*s*_*t* denotes the number of shortest paths connecting *s* and *t*.

## 4. Results

### 4.1. Subjective report results

#### 4.1.1. Reliability and validity

##### 4.1.1.1. Reliability

In this study, Cronbach's Alpha coefficient was used to analyze the reliability. All questionnaires completed by the 20 subjects in the trials were treated as independent samples, and the overall comfort and the eight single influences on comfort were examined as a set of questions. The results are shown in Table 2.

**Table 2:** Results of intrinsic reliability analysis of subjective comfort questionnaire.

	**Overall** **cronbach's** **alpha value**	**Corrected question and** **total correlation** **coefficients**	**Cronbach's alpha value** **after removing** **the question**
Overall comfort	0.814	0.637	0.778
Emotional valence		0.634	0.782
Emotional arousal		0.340	0.815
Static comfort		0.633	0.779
Noise comfort		0.513	0.795
Vibration comfort		0.584	0.789
Aural pressure comfort		0.526	0.794
Visual comfort		0.349	0.814
Thermal comfort		0.424	0.806

According to Table 2, the overall Cronbach's alpha value was higher than 0.7, indicating that the subjective comfort questionnaire has good reliability and that the overall comfort and the eight single influencing factors of comfort have a high degree of internal consistency and reliable findings. Except for visual comfort, all other questions caused a decrease in the overall Cronbach's Alpha when removed, while the correlation coefficient between visual comfort and the composition of the other questions is still higher than 0.3, so no questions are needed to be removed.

##### 4.1.1.2. Validity

Exploratory factor analysis was used to examine the validity of the nine questions, and factor rotation was performed using the maximum variance method. The analysis resulted in a KMO coefficient of 0.796, with a significance of less than 0.05, demonstrating that the data were well-suited to the use of exploratory factor tests. Each question had a load higher than 0.5 in at least one component, and therefore each question established its validity. The rotated component matrices are shown in Table 3.

**Table 3:** Component matrix after rotation.

**Question**	**Correlation coefficient with other questions**	**Cronbach's alpha value after removing the question**
Noise comfort	0.915	*
Aural pressure comfort	0.798	*
Thermal comfort	0.794	*
Vibration comfort	0.780	*
Visual comfort	0.558	*
Overall comfort	*	0.870
Static comfort	*	0.857
Emotional valence	*	0.851
Emotional arousal	*	0.764

*Means coefficient < 0.5.

**Figure 3:**
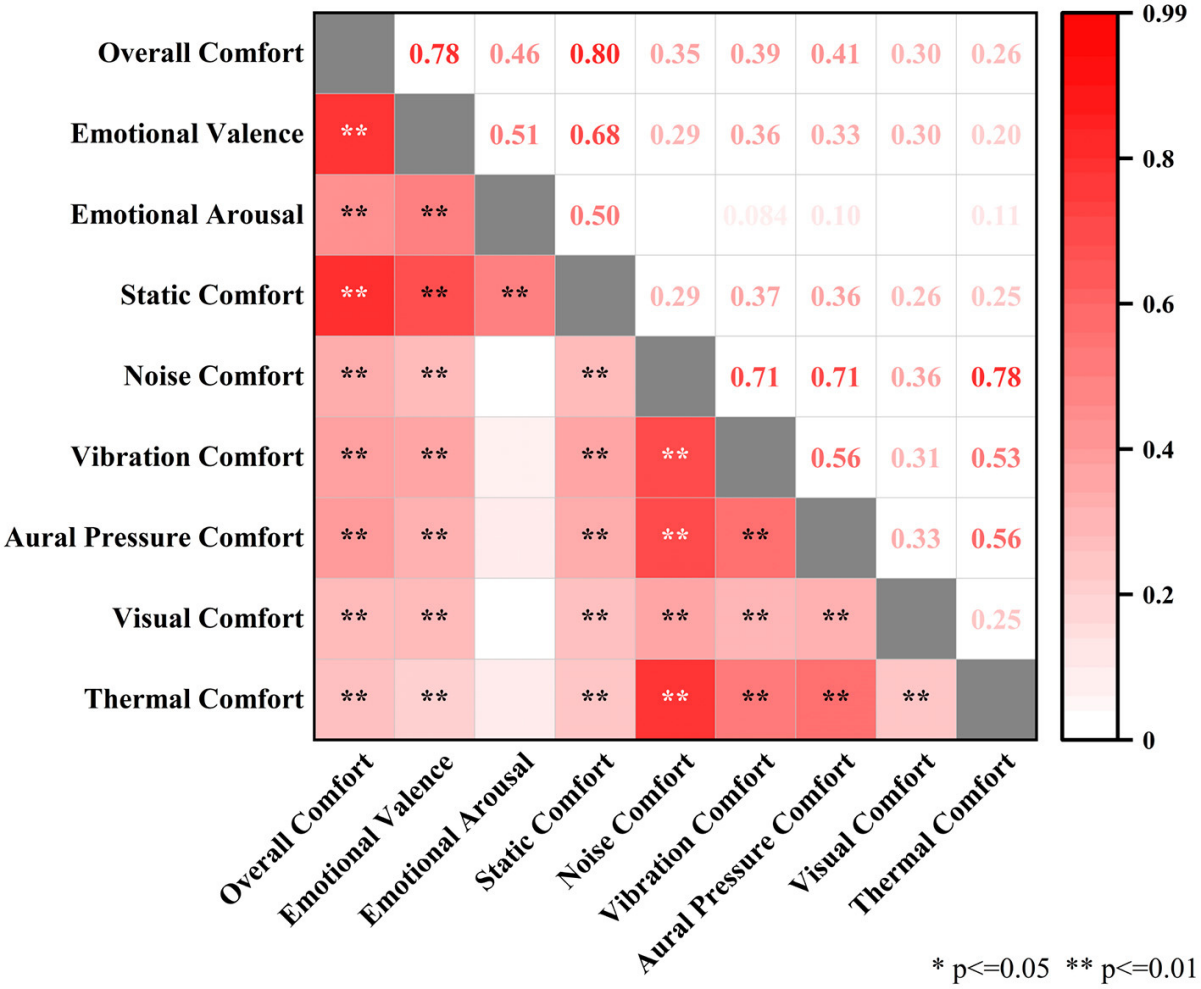
Overall comfort and eight single-influence comfort correlations.

Spearman's correlation coefficient was used for correlation analysis, and the results are shown in Figure 3. The top right corner represents the correlation coefficients between two variables, and the bottom right corner represents the *p*-value between two variables. *p*-values higher than 0.05 will not be shown in the graph. There is a correlation between the overall comfort and the subjective emotion of the passengers, as well as between the comfort of each environmental variable and the overall comfort, and between the comfort of each environmental variable.

In terms of correlation coefficients, the correlation coefficient between overall comfort and static comfort is the largest at 0.80, followed by the correlation coefficient with the subjective emotional valence of the passengers at 0.78. And emotional arousal, aural pressure comfort, vibration comfort, and noise comfort with correlation coefficients of 0.46, 0.41, 0.39, and 0.35, respectively. The overall comfort, light comfort, and thermal comfort can be regarded as uncorrelated. The correlation analysis discovers that overall comfort is strongly positively correlated with subjective emotional valence and static comfort of the subjects, while it is low positively correlated with emotional arousal, noise comfort, vibration comfort and aural pressure comfort. And overall comfort is not correlated with visual comfort and thermal comfort. This correlation can be seen as there is a similar change trend between static comfort and overall comfort.

### 4.2. EEG source connectivity results

In our previous work, we have identified the primary comfort-related rhythmic wave as the beta band. Therefore our research in this paper focuses on the EEG signal of the beta wave. In order to find the division of labor and synergy between brain regions when the HSR passengers feel uncomfortable, the source functional brain network was constructed by dividing the cerebral cortex into 95 brain regions, and their functional connectivities were calculated. Then the global and local attribute indices of the brain network were extracted. A power-free graph was constructed based on each subject's 95 ÃŮ 95 fully connected functional connectivity matrix and a choice was made to process all fully connected functional connectivity matrices by setting different sparsity to control the threshold. This paper uses a new sparsity range determination method proposed by (45). The sparsity range was determined to be 10–38% and eight functional brain networks were created at each sparsity level, i.e., 10, 14, 18, 22, 26, 30, 34, and 38%, when a growth step of 4% was chosen. In addition, in order to comprehensively compare the differences between functional brain networks of different sparsity, AUC was used as an overall evaluation index.

#### 4.2.1. Global attributes index at different sparse density

##### 4.2.1.1. Clustering coefficient (CC)

**Figure 4:**
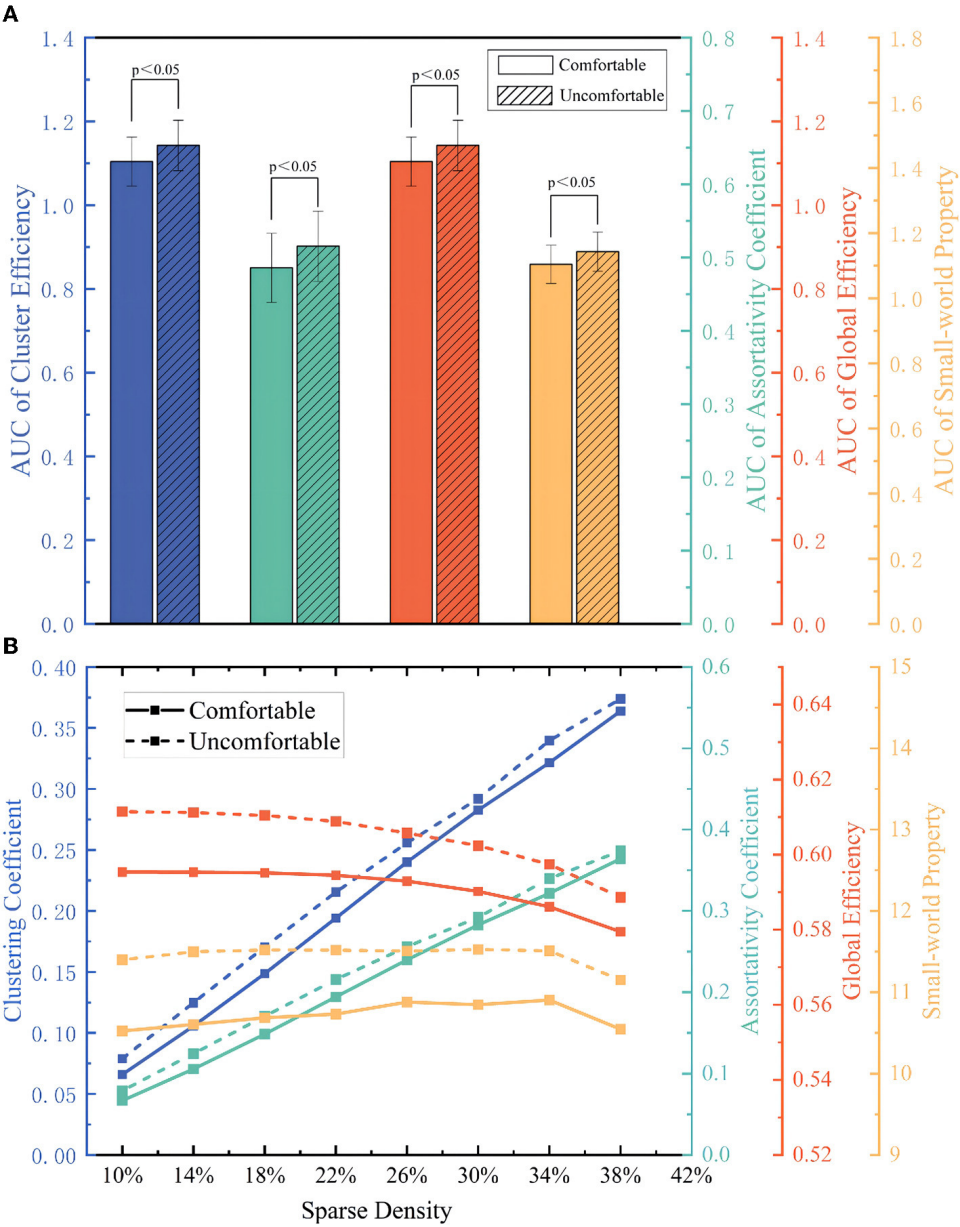
**(A)** Global attributes index at different sparse density; **(B)** The AUC of global attributes index.

CC is generally used to describe the integration function of the functional brain network. CC for the uncomfortable state was higher than that for the comfortable state for the whole range of sparse density and both of them increased with increasing sparse density. The results were shown in Figure 4A blue line. In addition, a paired *t*-test was carried out for a comparison of the AUC of comfortable passengers' brain network CC and that of uncomfortable passengers' brain network CC. As the Figure 4 blue bar shown, the CC for the uncomfortable brain network was significantly greater than comfortable one (*p* < 0.05).

##### 4.2.1.2. Assortativity coefficient (AC)

AC generally describes the tendency of nodes to connect to other nodes with a similar number of edges. Identical to CC, functional brain network' AC in the comfortable and uncomfortable states increased with increasing sparse density (Figure 4A, green line). The results of the paired *t*-test for the AUC were shown in Figure 4B green bars, which demonstrated that there was a significant difference between different states (*p* < 0.05).

##### 4.2.1.3. Global efficiency (GE)

As Figure 4A orange lines and orange bars shown, in the sparsity range of 10% to 38%, the global efficiency GE of the subject in both the comfortable and uncomfortable states decreased as the sparse density increased. The AUC of the global efficiency GE for the uncomfortable condition was significantly greater than the AUC value of the clustering coefficient for the comfortable state (*p* < 0.05).

##### 4.2.1.4. Small-world property (σ)

Networks with high σ are generally more resistant to attack and exhibit higher information transfer speeds, computational power and synchronization. As shown in Figure 4A yellow lines and yellow bars, in the sparsity range of 10–38%, as the sparse density increased, σ of the passengers in the comfortable and uncomfortable states first increased and then decreased. And σ of uncomfortable passengers' functional brain net was higher than that of the comfortable passengers. Naturally, the AUC of the σ in the uncomfortable state was significantly greater than σ in the comfortable state (*p* < 0.05).

#### 4.2.2. Local property results

##### 4.2.2.1. Node degree (Deg)

In this paper, the AUC of 95 brain nodes' Deg were calculated for comfortable passengers and uncomfortable passengers. The brain nodes which were significantly different between the comfortable and uncomfortable states were identified by paired *t*-test (*p* < 0.05). The naming and delineation of brain nodes corresponding to brain regions were referenced from the Tzourio-Mazoyer method (52, 53). The key brain nodes information and corresponding *p*-values were shown in Table 4.

**Table 4:** The key brain nodes with significantly different Deg.

**ASL_ROI**	**Tzourio-Mazoyer**	**Tzourio-Mazoyer**	***P*-value**
	**name**	**code**
Lateral frontal	Frontal_Mid_R	2202	0.0160
Lateral frontal	Frontal_Inf_Oper_R	2302	0.0053
Hippocampus+	Hippocampus_R	4102	0.0253
Amygdala	
Hippocampus+	ParaHippocampal_R	4112	0.0481
Amygdala	
Parietal	Fusiform_R	5402	0.0205
Temporal	Temporal_Mid_R	8202	0.0500

##### 4.2.2.2. Betweenness centrality (BC)

Similar to Deg, AUC of BC were calculated for all the nodes of the functional brain network, and paired *t*-tests were used to identify nodes with significant differences (*p* < 0.05). The key brain nodes information and *p*-values were shown in Table 5.

**Table 5:** The key brain nodes with significantly different BC.

**ASL_ROI**	**Tzourio-Mazoyer**	**Tzourio-Mazoyer**	***P*-value**
	**name**	**code**
Lateral frontal	Frontal_Mid_R	2202	0.0132
Medial frontal	Frontal_Sup_Medial_R	2602	0.0319
Insula	Insula_L	3001	0.0352
Occipital	Occipital_Mid_R	5202	0.0287

## 5. Discussion

### 5.1. The influencing factors of overall comfort: Subjective report

The overall comfort of passengers can be influence by many factors, such as vibration, noise, thermal environment, air pressure, light, seat and passenger emotion. It is important for us to understand the rank of the above factors in the HSR passengers' overall comfort. From our statistics model of subjective comfort rating in field tests, static comfort, emotional value, emotional arousal, aural pressure comfort, vibration comfort and noise comfort have significant contributions to the overall comfort.

Among them, static comfort plays the most important role in the overall comfort of the passengers essentially the comfort of the seat design. The high-speed train is a long-distance vehicle and passengers spend more time sitting in their seats. The seat not only provides a comfortable sitting position for the body but also influences the accumulation rate of fatigue in the musculoskeletal area of the hips and back. Discomfort during prolonged sitting increases the risk of musculoskeletal disorders and causes several health problems (24). The vibration of the train seat has a direct impact on the passengers' experience and therefore on the overall comfort. Noise comfort and aural pressure comfort, both of which can also be referred to as ear comfort, cannot be ignored. The passage of high-speed trains through tunnels results in increasing transient pressure changes and aerodynamic noise in the tunnel (4).

Emotions and the overall comfort of passengers interact with each other. The human judgment of the environment is also influenced by other sensory factors of the human body, which are often related to the perception and subjective mood, and the individual's subjective evaluation of the riding environment may be influenced to some extent by their psychological state and subjective emotions. So it may be possible to induce positive emotions to improve the overall comfort of the passengers (13).

The overall comfort of HSR passengers is independent of visual and thermal comfort. They are both important factors in other transport. Continuous upgrading of high-speed trains has resulted in significant improvements to the lighting in the carriages, which provide the comfortable lighting environment. In terms of thermal comfort, the high airtightness of high-speed trains and the heating, ventilation, and air conditioning (HVAC) system allows comfortable temperatures to be maintained in the face of rapid changes in external temperatures.

All in all, the visual and thermal comfort of the HSR carriages are excellent, due to the excellent lighting and air conditioning systems. However, the design of the seats still leaves something to be desired and does not provide passengers with good static comfort. In addition, emotional valence contributes significantly to overall comfort, and although a causal relationship is not clear, it is worth investigating the improvement of overall passenger comfort through the regulation of emotions.

### 5.2. The influencing mechanisms of overall comfort: Functional brain network analysis

#### 5.2.1. Global attribute

The clustering coefficient is generally used to describe the integration of functional brain networks. And the assortativity coefficient is used to describe the tendency of nodes to connect to other nodes with similar number of edges. As shown in Figure 4A, The CC and AC increased with increasing sparse density due to the increased number of connected edges in the brain network. Considering the AUC for the entire sparsity interval, the CC and the AC were significantly smaller for the subject in the comfortable state than in the uncomfortable one. This means that the brain connectivity of passengers in the uncomfortable state have more links than in the comfortable state. Previous findings (54) show that when subjects are in a stressful and anxious state, the connections between brain regions are more substantial, and the increase in CC and AC may indicate that passengers are more likely to experience anxiety when they are in an uncomfortable state.

As the sparse density increases, the edges of the brain network increase and the network becomes progressively more complex, which cause lower GE. GE is an attribute that measures the efficiency of information exchange in brain networks. As shown in Figure 4B, considering the full range of sparsity, the AUC of GE is significantly greater for the uncomfortable state than for the comfortable state. This may be attributed to the fact that passengers who are uncomfortable activate higher cognitive systems thus increasing network efficiency. When passengers feel uncomfortable, the functional segregation within the brain is lost, and the brain network shifts to a more random sleep-free network to ensure proper task performance, then leading to an increase in GE (55).

Unlike the above global attributes indices, the σ first increased and then decreased, as shown in Figure 4A. Specifically, in increasing a certain degree of sparsity, the information transmission speed of the train passengers' brain network became higher because of the increased edges. Still, when a certain number of edges were reached, the brain network became more invalid connections, the network function separation and function integration of the brain network could not be balanced, and the efficiency decreased. Subjects in a comfortable state, with a more relaxed mental state, have self-organized brain activity in a 'small world' network, through which the human brain can maintain more efficient communication in global brain regions with lower energy consumption (56).

#### 5.2.2. Local attribute

**Figure 5:**
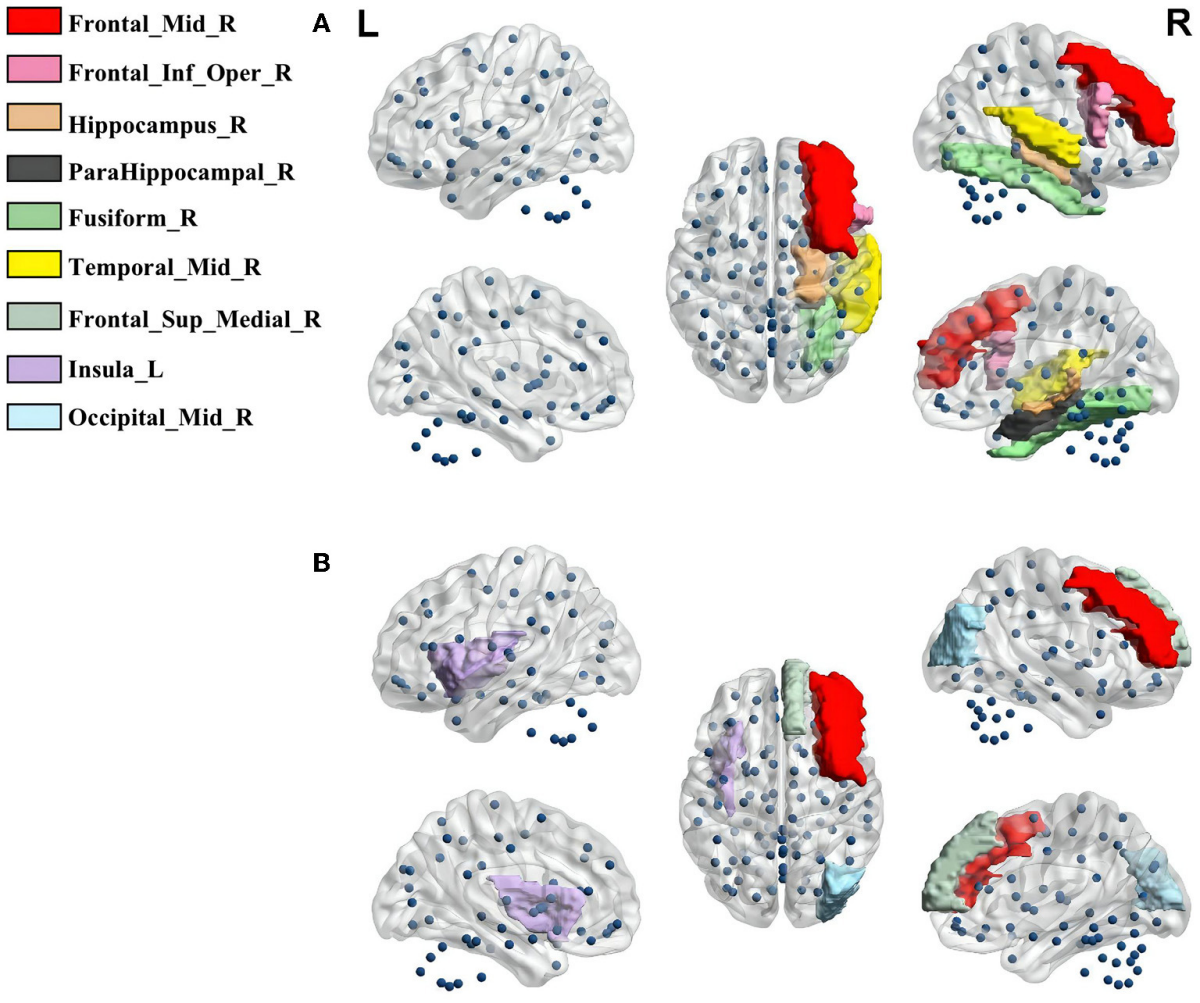
**(A)** The key brain nodes with significantly different Deg; **(B)** The key brain nodes with significantly different BC.

The node degree and betweenness centrality are generally used to describe the importance of that node in the brain network. A paired *t*-test revealed nine brain regions with significantly different local attribute (*p* < 0.05) between comfort and discomfort for the 20 subjects in the Beta band. Most of the nodes were all located in the right side of the brain, further demonstrating the laterality of the brain at different levels of comfort (Figure 5). Specifically, Brain Insula_L located in the frontal lobe of the brain together with Frontal_Mid_R, Frontal_Inf_Oper_R, and Frontal_Sup_Medial_R. They are involved in the emotional assessment of human, feeling the stress that accompanies discomfort (57). In addition, Fusiform_R and Temporal_Mid_R are located near the temporal lobe of the brain and are closely related to the control of emotions in humans (58). While Hippocampus_R and ParaHippocampal_R are located in the Hippocampus and Amygdala of the brain, which are mainly responsible for spatial perception and emotion of the human body (59). These nodes, which vary significantly in the brain network, confirm that emotion and spatial perception are key to comfort.

## 6. Conclusions

This paper revealed influencing factors and mechanisms of HSR passenger overall comfort by using subjective reports and source functional brain network through a series of field ergonomics tests. The results of the subjective report showed that the overall comfort of the HSR passengers was most influenced by static comfort, followed by the passengers's own emotional valence, emotional arousal, aural pressure comfort, vibration comfort, and noise comfort. Visual comfort and thermal comfort having less influence on the overall comfort. Combining ESI and functional connectivity computational methods, the source functional brain networks of the HSR passengers in the comfort and discomfort states were constructed and statistically compared for analysis. The results revealed that the higher synchronization and shorter transmission paths between brain regions in the discomfort state led to higher global efficiency. And there would be more obvious clustering effects between brain regions. The key brain regions were found by local attribute indices, demonstrating the key role of emotion and spatial perception in the discomfort state. The results of both the subjective reports and the analysis of the functional brain network give evidence of emotion is an important factor influencing comfort. The methods of improving comfort through emotion regulation and seat design are worthy of further research.

## Data availability statement

The datasets presented in this article are not readily available because the datasets are being further analyzed. Requests to access the datasets should be directed to the corresponding author.

## Ethics statement

The studies involving human participants were reviewed and approved by Xiangya No.2 Hospital of Central South University Institutional Review Board. The patients/participants provided their written informed consent to participate in this study.

## Author contributions

CF, YLin, and WZ carried out the experiment. CF and SL wrote the manuscript with support from YLi, FW, DZ, and XX. YP supervised the project. All authors contributed to the article and approved the submitted version.

## Funding

This study was supported by the National Natural Science Foundation of China (Grant Number: 52075553), the Human Science Foundation for Distinguished Young Scholars of China (Grant Number: 2021JJ10059), the Postgraduate Scientific Research Innovation Project of Hunan Province (Grant Number: CX20210099), Human Factors and Ergonomics Industry-University Cooperation Collaborative Education Project of Higher Education Department of Ministry of Education of the People's Republic of China (Grant Number: 202107ZCJG05), and Technical Service Projects of China Railway Signal & Communication Corporation Limited (Grant Number: 2300-K1200035.04).

## Conflict of interest

The authors declare that the research was conducted in the absence of any commercial or financial relationships that could be construed as a potential conflict of interest.

## Publisher's note

All claims expressed in this article are solely those of the authors and do not necessarily represent those of their affiliated organizations, or those of the publisher, the editors and the reviewers. Any product that may be evaluated in this article, or claim that may be made by its manufacturer, is not guaranteed or endorsed by the publisher.
